# Central Line Guidewire Knot in a Paediatric Patient with Bronchial Leiomyosarcoma Undergoing Left Pneumonectomy: A Case Report

**DOI:** 10.4274/TJAR.2025.252060

**Published:** 2025-12-22

**Authors:** Suruchi Richhariya, Sunaina Tejpal Karna, Pramod Kumar Sharma, Roshan Chanchalani

**Affiliations:** 1 All India Institute of Medical Sciences Bhopal, Department of Anaesthesiology, Madhya Pradesh, India; 2 All India Institute of Medical Sciences Bhopal, Department of Paediatric Surgery, Madhya Pradesh, India

**Keywords:** Guidewire knotting, internal jugular vein catheterisation, one-lung ventilation, paediatric anaesthesia, ultrasound-guided central venous catheterization

## Abstract

We report the unanticipated intraoperative complication of a guidewire knot during central venous line insertion in the left internal jugular vein (IJV), in a child scheduled for a left pneumonectomy for leiomyosarcoma of the left lung under general anaesthesia. After an uneventful guidewire placement in the left IJV under ultrasound guidance, difficulty was encountered in advancing the central venous catheter over the guidewire. Resistance was felt when initiating the removal of the guidewire. The guidewire knot was identified with intraoperative fluoroscopic imaging. After consultation with the surgical team, the knot in the guidewire was removed by immediate venotomy. Intraoperative lung isolation and tracheal extubation after the surgery were uneventful. This report emphasises the importance of vigilance during central venous catheterisation in paediatric patients whose anatomical variations and smaller vessels exacerbate the risk of such complications. Ultrasound-based preprocedural Rapid Central Venous Assessment, and intra-procedural guidewire-tip navigation may help prevent coiling/knotting. Furthermore, it highlights the need for rapid recognition and surgical readiness to resolve unexpected issues during routine procedures.

**Main Points**

• A knot in a central line guidewire can occur in the distal internal jugular vein in paediatric patients with distorted anatomy despite ultrasound guidance with an out-of-plane approach.

• Pre-procedural ultrasound-guided Rapid Central Venous assessment and intraprocedural tip navigation may help to avoid guidewire coiling and knotting.

• Resistance during guidewire removal may indicate knotting, especially if the guidewire coils around vessel walls.

• Blind, forceful removal must be avoided as it may lead to tissue entrapment and damage.

• The key to a successful outcome is prompt recognition of this complication and individualised management according to the scenario and available resources.

## Introduction

We share the challenges faced in the anaesthetic management of a child scheduled for left pneumonectomy for leiomyosarcoma of the lung, and the complications encountered with subsequent management. This case underscores the need for vigilance during central venous catheterisation in paediatric patients.

## Case Report

**Figure 1:**
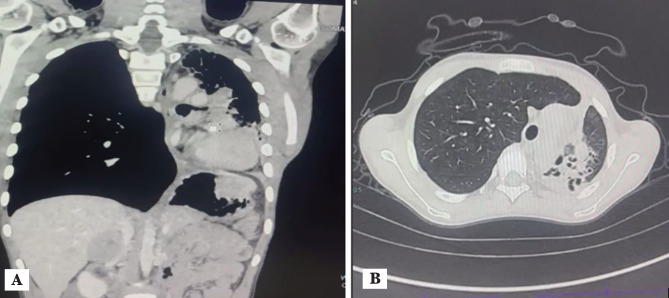
Contrast enhanced computed tomography (CECT) showing mediastinal shift to the left side. A) Sagittal view; B) Coronal view.

A 10-year-old child weighing 20 kg, with a body mass index of 11.3 kg m^2^, presented to the paediatric outpatient department of our institute with cough and expectoration for 6 months, and shortness of breath for 2 weeks. The child was accompanied by his father. Pre-operative contrast-enhanced computed tomography (CT) suggested a large, ill-defined, heterogeneously enhancing lesion in the posteroinferior aspect of the left side of the thoracic cavity. The lesion infiltrated the left main bronchus, completely occluding the lower lobe and partially occluding the upper lobe. Furthermore, the lesion caused a mediastinal shift to the left and was associated with abdominal metastasis, which was confirmed as a spindle cell neoplasm/leiomyoma on CT-guided biopsy (Figure 1). Pulmonary function tests showed borderline airway obstruction, which improved on bronchodilator therapy. During the pre-operative assessment, the child complained of pain on deep inspiration, and decreased air entry was observed in the left lung. The rest of the general and systemic examinations were unremarkable. The pulmonologist advised peri-operative bronchodilator nebulisation. The parents provided written informed consent for surgery and general anaesthesia, epidural catheterisation, invasive central and arterial line insertion, one-lung ventilation, post-operative mechanical ventilation, and intensive care.

The right-sided double-lumen tube (DLT) size was estimated pre-operatively on a CT scan based on the size of the trachea and the right and left bronchi.

**Figure 2:**
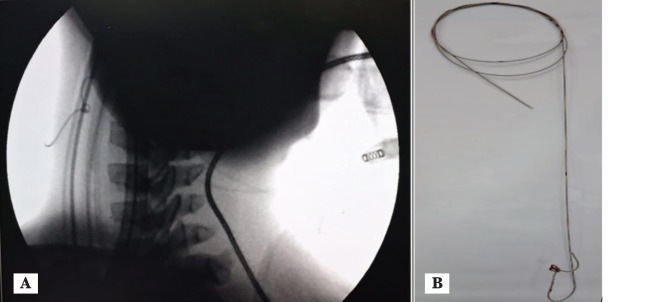
The central line guidewire knot: A) Fluoroscopic view of central line guidewire knot in left internal jugular vein; B) The knotted central line guidewire after surgical extraction.

On the day of the surgery, after the uneventful induction of anaesthesia, a 28 Fr right-sided DLT was placed under direct laryngoscopic vision and confirmed with a paediatric fibreoptic bronchoscope. Sevoflurane with an air-oxygen mixture was used for anaesthesia maintenance, with a 0.5 mg kg^-1^ h^-1^ infusion of atracurium for muscle relaxation. Analgesia was maintained with an epidural infusion of 0.125% levobupivacaine with 1 µg mL^-1^ fentanyl through an 18G epidural catheter inserted at the T6-T7 level. The surgery was performed in the right lateral position. The plan was to insert an ultrasound-guided, left internal jugular vein (IJV) 5.0 French triple-lumen central venous catheter of 8 cm length. The needle was placed in the left IJV in a single attempt using an out-of-plane approach, and the guidewire was passed without resistance through the needle up to a sufficient length of 15 cm. After dilating the subcutaneous tract with a dilator, the triple-lumen catheter was railroaded over the guidewire; however, it failed to pass beyond 4 cm. Resistance was felt when attempting to remove the guidewire. Ultrasonography confirmed that the guidewire was present in the IJV lumen. Fluoroscopy performed in the operating theatre with the C-arm showed a knot in the guidewire (Figure 2).

As the patient was under general anaesthesia in the operating theatre, exploration of the IJV to remove the guidewire knot was planned in discussion with the surgical team. Venotomy was performed under aseptic conditions, and the guidewire knot was observed to be in the wall of the vein. The knot was removed after dilating the venotomy, and the site was sutured. A 5 French femoral central line was placed in the right femoral vein.

After thoracotomy, the child tolerated one-lung ventilation well, with no episodes of desaturation. During the separation of the adhesions between the lung and precordium, norepinephrine infusion was titrated to correct hypotension.

Electrolytes and blood gas parameters were monitored and corrected with serial arterial blood gas analysis. The urine output was 0.5-1 mL kg^-1^ h^-1^ throughout the surgery. After ensuring an adequate margin from the carina, the paediatric surgical team performed a left pneumonectomy. The tracheal and bronchial lumen of the DLT was thoroughly suctioned, and the bronchial cuff was deflated. The DLT was removed after ensuring that no bleeding or tissue debris was present in the carina, using a check bronchoscopy. A cuffed polyvinyl chloride endotracheal tube of size 5.5 was inserted into the tracheal lumen under video laryngoscopic guidance.

The neuromuscular blockade was reversed after adequate, pain-free respiratory efforts, and the trachea was extubated. Analgesia was sufficiently maintained via patient-controlled epidural infusion of 0.1% bupivacaine with 1 µg mL^-1^ fentanyl at a background infusion rate of 3 mL h^-1^, with a demand dose of 3 mL, a maximum of 3 doses per hour, and a lockout interval of 10 minutes. The patient’s post-operative recovery was good.

## Discussion

When performing left pneumonectomy in a 10-year-old child, the primary anaesthetic concern is achieving good lung isolation to facilitate surgery and manage intraoperative oxygenation and haemodynamics. Leiomyosarcoma is a specific type of malignant spindle cell neoplasm arising from the lower respiratory tract. This tumour originates from the smooth muscle and is rare in paediatric populations, with only a few cases reported in the literature.^1, 2^ There are certain unique considerations specific to anaesthesia management in a paediatric patient, with left bronchial leiomyosarcoma, scheduled for left pneumonectomy. These concerns are related to the multidisciplinary pre-operative assessment to select the optimal technique for one-lung ventilation, hemodynamic monitoring, peri-operative analgesia, and fast-tracking of anaesthesia. We have discussed the complications of the guidewire knot encountered in the placement of the central venous catheter in the left IJV and its related preventive and management strategies.

A systematic, standardised approach like Rapid Central Venous Assessment before central venous catheterisation may help identify anatomical alterations or thrombus in previously catheterised veins. Rapid evaluation of the six central veins in the supra/infraclavicular area can help to rule out abnormalities like thrombosis, stenosis, external compression, anatomical variation, and to choose an appropriate catheter vein size (1:3 or less).^3^ This is important, as guidewire coiling and knotting are often reported when re-catheterising a previously catheterised vein or during thrombosis. These complications can be both extravascular and intravascular.^4, 5^ Though we inserted the guide wire using the out-of-plane approach, it may have been prudent to navigate the tip’s direction using the in-plane approach as part of the “SIC” protocol (Safe Insertion of Central Catheters).^6^ Immediately after ultrasound-guided venipuncture, ultrasound should be used to assess the correct direction of the guidewire (ultrasound-based tip navigation) by scanning the veins of the supraclavicular region with the same linear probe used for needle insertion.^6^ In a paediatric patient, using a hockey stick probe may facilitate this. However, a micro-introducer kit with a 21G echogenic needle and a 0.018” nitinol guidewire with a straight soft tip, to allow tip navigation, is recommended, especially in infraclavicular venipunctures. An intracavitary electroencephalogram is the most cost-effective and accurate intraprocedural method for determining the tip location.^7^

The tensile strength of guidewires is limited, and excessive force during insertion can easily cause coiling. Upon removal, this coiling can tighten into a knot. These issues are often faced in subclavian vein cannulation owing to its non-linear course, predisposing the guidewire to coil or loop over the first rib. Complications such as guidewire breakage, impaction and paradoxical embolisation of fractured wire segments have also been recorded.^5, 8^ Guidewire kinking has been noted more frequently in the paediatric population, especially younger children.^9^

While knotting is more commonly associated with subclavian vein cannulations, reports of knots in the femoral vein exist.^10^ Applying force to remove a knotted guidewire should be avoided, as it can lead to shearing of the inner and outer coils, resulting in severe vascular injury if the coil and knot are intravascular.^11^ A knotted guidewire should be removed under fluoroscopic guidance when available or via open surgical exploration.

Knotting during left IJV cannulation has also been reported, especially in cases where anatomical structures are altered, as was observed in our patient.^12^ Potential complications should be anticipated at any stage of central line insertion.

## Conclusion

Besides ultrasound-guided guidewire insertion, real-time or post-advancement navigation of the guidewire through the needle throughout the vein (up until the superior vena cava for the neck veins) is a crucial step which may help avoid coiling and subsequent knotting of the guidewire at the time of insertion. Prompt recognition, prevention of unnecessary force on removal, and surgical intervention can rectify the complication, enabling the pneumonectomy to progress smoothly. This case offers valuable insights for emerging anaesthesiologists, highlighting the significance of awareness and preparedness for unexpected complications.

## Ethics

**Informed Consent:** The parents provided written informed consent for surgery and general anaesthesia, epidural catheterisation, invasive central and arterial line insertion, one-lung ventilation, post-operative mechanical ventilation, and intensive care.

## References

[1] Parham DM, Alaggio R, Coffin CM (2012). Myogenic tumors in children and adolescents.. Pediatr Dev Pathol.

[2] Orman G, Masand P, Hicks J, Huisman TAGM, Guillerman RP (2020). Pediatric thoracic mass lesions: beyond the common.. Eur J Radiol Open.

[3] Spencer TR, Pittiruti M (2019). Rapid Central Vein Assessment (RaCeVA): a systematic, standardized approach for ultrasound assessment before central venous catheterization.. J Vasc Access.

[4] Wang LP, Einarsson E (1987). A complication of subclavian vein catheterisation. Extravascular knotting of a guidewire.. Acta Anaesthesiol Scand.

[5] Lee JJ, Kim JS, Jeong WS, Kim DY, Hwang SM, Lim SY (2010). A complication of subclavian venous catheterization: extravascular kinking, knotting, and entrapment of the guidewire -a case report-.. Korean J Anesthesiol.

[6] Brescia F, Pittiruti M, Ostroff M, Spencer TR, Dawson RB (2023). The SIC protocol: a seven-step strategy to minimize complications potentially related to the insertion of centrally inserted central catheters.. J Vasc Access.

[7] Pittiruti M, Pelagatti F, Pinelli F (2021). Intracavitary electrocardiography for tip location during central venous catheterization: a narrative review of 70 years of clinical studies.. J Vasc Access.

[8] Casserly IP, Goldstein JA, Rogers JH, Lasala JM (2002). Paradoxical embolization of a fractured guidewire: successful retrieval from left atrium using a snare device.. Cathet Cardio Intervent.

[9] Lamsal R, Dube SK, Bindra A (2018). Difficulty in central venous cannulation due to kinked guidewire: intravenous cannula comes to rescue!. J Neuroanaesth Crit Care.

[10] Khan KZ, Graham D, Ermenyi A, Pillay WR (2007). Case report: managing a knotted Seldinger wire in the subclavian vein during central venous cannulation.. Can J Anaesth.

[11] Sekiguchi K, Takano H (2022). Knot formation of a guidewire during subclavian venous catheterization: a case report.. Radiology Case Reports.

[12] Pal RK, Laha B, Nandy S, Biswas R (2014). Coiling of guide wire in the internal jugular vein during central venous catheter insertion: a rare complication.. Indian J Anaesth.

